# Biostimulants efficacy in growing *Sonchus oleraceus* plants in contaminated mining soil with potentially toxic elements (PTEs)

**DOI:** 10.1007/s11356-025-36577-z

**Published:** 2025-06-09

**Authors:** Aspasia Grammenou, Spyridon A. Petropoulos, Georgios Thalassinos, Vasileios Antoniadis

**Affiliations:** Department of Agriculture Crop Production and Rural Environment, University of Thessaly, Fytokou Street, 384 46, Vólos, Greece

**Keywords:** *Sonchus oleraceus*, Biostimulants, Phytoremediation, Contaminated soils, Soil health, Potentially toxic elements

## Abstract

Evaluating the effects of innovative amendments on potentially toxic element (PTE) availability is essential for managing contaminated soils and enhancing agricultural productivity. In the present work, we investigated the mitigation capacity of various biostimulant formulations (*Trichoderma* sp., Phosbactin, *Bacillus* sp., Azospir, and humic substances) in *Sonchus oleraceus* grown in a severely contaminated soil with potentially toxic elements (PTEs) from the Lavrio mining area in Greece. We aimed to determine the impact of biostimulants on PTE mobility in soil and their accumulation in plant tissues. For this purpose, we conducted a pot experiment with seven treatments, i.e., non-contaminated soil (negative control), contaminated soil from Lavrio (positive control; PC), and five biostimulant treatments added in the Lavrio soil, resulting in 105 pots (7 treatments × 15 replicates). All treatments were fertigated with nutrient solution (200 mg L^−1^ of N-P-K) at regular intervals. Significant variations in *S. oleraceus* growth and PTE concentrations were observed. The Lavrio soil (positive control) showed reduced plant growth parameters compared to the negative control. Specifically, the average plant height notably decreased from 21.63 cm to 15.92 cm, while the fresh biomass dropped by 60.4% (from 15.50 g to 6.14 g), indicating a negative impact on plant growth processes. The application of biostimulants showed no notable differences on growth parameters, except for the reduced plant height compared to the non-contaminated soil (negative control). Certain biostimulants, like Phosbactin, Azospir, *Bacillus* spp. (BAC), and humic and fulvic acids, tended to cause an increase in the DTPA extractability of Pb, Cu, and Zn, although only BAC caused a significant increase in Zn (589.92 mg kg^−1^) compared to the PC (381.20 mg kg^−1^). Moreover, *S. oleraceus* accumulated increased PTE concentrations, particularly Cd, Zn and Pb. The highest concentration of Cd was found in the roots, reaching 39.42 mg kg^−1^ at AZO, while Zn concentration peaked at 2911.99 mg kg^−1^ at PH. The bioaccumulation factors (BAF) indicated a high uptake of Cd and Zn, with values of 5.831 and 3.221, respectively. Pb exhibited the highest mobility, with a transfer factor (TF) exceeding 1, especially at AZO (TF = 2.817). In contrast, Cd and Zn demonstrated limited translocation potential (TF < 0.5). *S. oleraceus* displayed a hyperaccumulator behavior, despite limited translocation to aerial parts, making it potentially suitable for phytoremediation of contaminated soils. These findings demonstrate the complex interactions among biostimulants, soil, plant responses and PTE concentrations, highlighting the importance of choosing suitable biostimulants for the sustainable management of PTE-contaminated soils. Further investigation is needed to identify biostimulant mechanisms mitigating PTE negative effects.

**Supplementary information:**

The online version contains supplementary material available at 10.1007/s11356-025-36577-z.

## Introduction

A major issue for crop production, human health, and the environment is tο understand the behavior of potentially toxic elements (PTEs) and their toxic effects on plants (Thalassinos et al. 2023b). Although the origin of PTEs is both natural and anthropogenic, their elevated concentrations recorded in the last decades are mainly due to anthropogenic activities (Antoniadis et al. 2022). Metal mining has been a major anthropogenic source of PTEs released into neighboring areas of mining sites (Mensah and Addai 2024). Long-term mining and smelting activities in the region of Lavrio, located in southeastern Greece, particularly in zinc (Zn) and lead (Pb) ores, have contributed to substantial environmental degradation of the region due to high levels of PTEs (Pappa et al. 2018; Antoniadis et al. 2022). PTEs such as cadmium (Cd), Pb, copper (Cu), and Zn have been reported in the literature for the region of Lavrio (Kalyvas et al. 2018, 2022). These PTEs are persistent pollutants due to their non-degradable nature, posing long-term risks to natural habitats and human health (Alam et al. 2024).

One of the major challenges of the last decades has been the management of contaminated soils for restoring soil quality and ensuring human health and safety. Numerous remediation practices, categorized into physicochemical and biological methods, have been utilized to mitigate PTEs toxicity and reclaim contaminated sites (Palansooriya et al. 2020). Physicochemical approaches such as soil washing, solidification, and electro-reclamation offer rapid but often costly solutions, while bioremediation, including microbial remediation, phytoremediation and bioleaching, is considered an eco-friendly accepted alternative for long-term soil restoration (Zheng et al. 2024a). Among these, phytoremediation stands out as a sustainable approach that relies on using plants to remediate elevated PTE concentrations in soils without impairing soil structure (Antoniadis et al. 2021). A variety of indigenous plant species, also referred to as metallophytes, may naturally grow in metal-rich soils. These plants have developed unique adaptation mechanisms that facilitate increased resistance and the uptake of PTEs in contaminated soils (Kikis et al. 2024). Such properties meet the standard conditions of metal hyperaccumulation, which refers to the ability of plants to relocate metals from soil to the above-ground biomass at a high rate (Kikis et al. 2024).

The success of phytoremediation depends on the selection of plant species capable of accumulating and tolerating high levels of PTEs (Kikis et al. 2024). In this context, *Sonchus oleraceus*, commonly known as “sowthistle,” is an herbaceous wild edible plant found in varied climate and soil conditions, including environments characterized by high levels of PTEs (Peerzada et al. 2019; Sánchez-Aguirre et al. 2024). According to the literature, its fast growth and efficiency to accumulate and translocate PTEs, such as Pb and Cd, makes it a candidate species to be included in phytoremediation strategies Grammenou et al. 2023).

In an effort to enhance the phytoremediation potential of plants, the use of biostimulants has gained attention. Biostimulants are a promising tool in modern sustainable agriculture. They are substances of varied composition that may enhance plant development through the induction of various mechanisms (Shahrajabian et al. 2023). These innovative amendments have also gathered the attention of scientific community for their ability to enhance plant tolerance to adverse conditions (Grammenou et al. 2023). These products, which may include microbial inoculants (e.g., *Trichoderma* spp., *Bacillus* spp., phosphate solubilizing bacteria (PSB)) and organic amendments such as humic and fulvic acids, have shown a high potential to improve soil health by reducing the availability of PTEs in soil (Dogan et al. 2022). Biostimulants are an eco-friendly alternative to synthetic chelating agents, such as EDTA, as they form complexes with cationic metals, affecting their availability and mobility (Grammenou et al. 2023). The chelation process is crucial for releasing PTEs from solid matrices, allowing their conversion into more soluble forms. Chelating agents bind PTEs through multiple bonds, thus facilitating their removal from soil (Alam et al. 2024). Humic substances and microbial biostimulants can influence PTE fixation by forming complexes or interactions with microbial biomass. Previous studies have indicated that microalgae and bacteria showed high absorption capacity of PTEs (e.g., Zn^2+^, Cd^2+^) from aqueous systems (Zheng et al. 2024b). In addition, biostimulants have been acknowledged for their ability to alleviate PTE toxicity in plants through various mechanisms, such as phytochelatin synthesis, the induction of the antioxidant defense systems, and metal chelation (Grammenou et al. 2023). The use of suitable biostimulants can lead to low-cost soil restoration and follow the principles of green sustainable remediation strategies (Grammenou et al. 2024).

Despite the increasing interest in biostimulants and their role in modulating PTE uptake and translocation in hyperaccumulator or tolerant species, biostimulants have not been thoroughly investigated. To address this gap, the present study focused on the use of biostimulant products in *Sonchus oleraceus* plants grown in a heavily contaminated soil from the Lavrio mining area in Greece on the remediation of PTEs, specifically Cd, Pb, and Zn. The main objectives were: (1) to evaluate the efficacy of different biostimulants in influencing the mobility and accumulation of PTEs in soil and plants, (2) to understand the interactions between sowthistle and PTEs, and (3) to elucidate the mechanisms of biostimulants through which biostimulants affect PTE bioavailabity and enhance plant vitality. The findings of this research are expected to provide a significant contribution to the practical implementation of biostimulants as a sustainable agricultural practice in soils affected by PTE contamination. Thus, the results may support efforts to restore environmental quality and soil health, and to ensure food safety and human health.

## Materials and methods

### Experimental design

A 52-day pot experiment was established under greenhouse conditions using a non-contaminated soil (as negative control; NC), collected from the University Farm at Velestino (22.756 E, 39.395 N) and a contaminated soil collected from the mining region of Lavrio, South Attica, Greece (37.719311° Ν, 24.044154° Ε) (as positive control; PC). The soil derived from Velestino was a loam (with sand and clay contents of 45 and 16%, respectively), pH 7.8, organic C 1.1%, and pseudo-total concentrations of 0.8, 80.8, 42.44, and 89.03 mg kg^−1^ for Cd, Pb, Cu, and Zn, respectively. According to the World Reference Base (WRB), the soil was a Cambisol, with total N of 1198.3 mg kg^−1^ and C/N 9.18. The contaminated soil was a loamy sand (sand 79.6%, silt 11.6%, clay 8.8%), alkaline (pH 8.0), and with low levels of organic C (1.1%). The pseudo-total metal concentrations in this soil were 85.59, 19,232.7, 70.84, and 15,663 mg kg^−1^ for Cd, Pb, Cu, and Zn, respectively. These concentrations exceeded the safety thresholds established for agricultural and residential soils. The Lavrio soil was a Leptosol (as per WRB), with total N of 976.6 mg kg^−1^, and C/N 12.8. A mix of soil and perlite (3:1 ratio) was used to fill 2-L pots. Perlite was employed due to its aeration properties, while chemically it is totally inert. Twenty-day old seedlings of *Sonchus oleraceus* were transplanted into the pots. The experimental design consisted of the 2 control treatments mentioned above and 5 amendments in the Lavrio soil; each one represented a type of biostimulant. (Table S1; supplementary material). The biostimulants used in this experiment were as follows:

i. A solid consortium of *Bacillus* species (i.e., *B. megaerium*, *B. altitudiris*, *B. subtilis*, *B. licheniformis*, and *B. methylotrophicus*) containing 10^9^ cfu g^−1^ of each species. An amount of 0.6 g of the solid consortium was diluted in 900 mL of distilled water, creating a solution of pH = 6.57 and OC = 5.29%. Each pot of this treatment received 3 mL of this solution on the day of transplantation. On days 15 and 30, additional applications of 3 mL were administered.
ii. A liquid solution of *Trichoderma harzianum* T78. Each pot received 3 mL of this solution. Subsequently, during the cultivation period, applications of 3 mL on days 15 and 30 were added.
iii. Phosbactin: Α commercial product in liquid form with pH = 5.76 and EC = 68 μS cm^−1^, containing 1 × 10^12^ cfu L^−1^ of *Bacillus megaterium*. 0.192 mL of the product were diluted in 1.5 L distilled water and each pot received 100 mL of the final solution.
iv. Azospir, a commercial product in liquid form with pH = 5.6 and EC = 63 mS cm^−1^, containing 1 × 10^12^ *Azospirillum* sp. and 1 × 10^12^ cfu L^−1^ *Azotobacter* sp. 0.384 mL of this product were diluted in 288 mL distilled water and each pot received 100 mL of the final solution.
v. A stock solution of humic and fulvic acids (70:30 ratio) derived from refined leonardite extract, with a pH of 8.53 and organic carbon (OC) equal to 4.83%. Stock solution was diluted by adding 1.1 mL in 373.9 mL distilled water. After the dilution, the roots of the seedlings were dipped in this solution. Moreover, the biostimulant was added at 50 mL per pot on days 5, 15, 25 after transplantation.

All the tested biostimulants were applied through fertigation with direct application of the implemented formulations in the soil. Moreover, all plants were supplied with a nutrient solution containing 200 mg L^−1^ of N-P-K tailored to the specific requirements of the plants. This solution was applied every 7 days at 100–150 mL per application. The duration of the experiment was extended until the flowering stage of the plants (harvest day 11 January 2024). The short-term duration of our experiment was due to the short growing period of the species. The aim of our study was to study the response of *S. oleaceus* to PTE toxicity and biostimulant application under controlled conditions which was conducted in pots in an unheated greenhouse. Under field conditions, unexpected rain could result in leaching of PTEs or could conceal the capacity of the tested biostimulants to mitigate any toxic effects of PTEs on the tested plants. Therefore, our results provide a preliminary evidence for the phytoremediative efficacy of the species.

### Soil and plant analyses

#### Pre-harvest measurements

Prior to harvest, plant growth characteristics were measured, including diameter of the rosette, height of plants, and number of leaves for all plants. For each treatment, chlorophyll content of the leaves was measured before harvest from the higher and most well-developed leaves using a portable chlorophyll meter (SPAD).

#### Plant growth assessment and chemical analyses

On harvest day, total fresh weight (g), total leaf area (cm^2^), and specific leaf area (SLA; m^2^ kg^−1^) were measured. Total leaf area was measured using a leaf area meter (LI-3100 C; LI-COR Biosciences; Hellamco S.A, Athens, Greece) and SLA was calculated as the total leaf area divided by the dry leaf weight. The aerial parts of plants (leaves and stems) were cut, and the roots were carefully removed by delicately rinsing the adhered soil particles. Aerial parts and roots were oven-dried (70 ℃) to constant weight. The experiment consisted of 15 replicates per treatment, which were then pooled into 5 composite samples, each for soil and plant samples. Soil samples were air-dried and analyzed for organic carbon content using the Walkely and Black method, and pH and EC were measured at 1:5 H_2_O. The pseudo-total concentration of PTEs in the soil was determined by aqua regia digestion (HNO_3_:HCl at ratio 1:3), and the bioavailable concentration of PTEs was extracted using DTPA-CaCl_2_ (1:2, soil:solution). Plant tissues were extracted for PTE levels by dry ashing (500 ℃, 5 h) and extraction with HCl (10 mL, 20%). The concentrations of Cd, Pb, Cu and Zn were determined by atomic absorption spectroscopy (Perkin Elmer A330). All methods described above followed the standard methodology described by Rowell (Rowell 1994).

#### Plant indices

Based on the primary data, indices were determined as per Kikis et al. (2024)

Bioaccumulation Factor (BAF) is defined as:

1 $$BAF=\frac{\text{Concentration of PTEs in aerial biomass}}{\text{DTPA}-\text{extractable PTEs in soil}}$$

Translocation Factor (TF) is defined as:

2 $$TF=\frac{\text{Concentration of PTEs in aerial biomass}}{Concentration of PTEs in roots}$$

### Quality control and data analysis

Data quality control involved the use of blanks in each extraction batch, achieving recovery rates from 92 to 108%, with all analyses performed in triplicates. The primary and secondary data underwent a one-way ANOVA (analysis of variance) to identify significant differences among treatments at 95% level (*p* < 0.05). Additionally, the Duncan’s post hoc analysis was used. Statistical analysis was performed by using the SPSS software (IBM SPSS Statistics v.29).

## Results

### Soil properties

The pH, EC, and organic carbon (OC) values of the tested soil showed statistically significant differences among the treatments (Table 1). As for pH, the soils of Velestino (non-contaminated as negative control; NC) and Lavrio (heavily contaminated but without biostimulant amendments as positive control; PC) had the highest pH (8.02 and 8.01, respectively), while the contaminated soil treated with AZO had the lowest pH (7.69). The pH values also decreased in the treatments of TR (7.84) and HFA (7.85) compared to the controls. Regarding EC, significant differences were recorded among the NC and PC, with PC showing lower values (108.82 μS cm^−1^), while the application of Azospir (AZO) led to the highest overall value of EC (227.48 μS cm^−1^). The OC content was significantly higher in the NC treatment (1.49%), while no significant variations were observed among the rest of the treatments.

**Table 1:** Soil properties (pH, EC and organic carbon) as affected by the biostimulant products

	**pH**	**EC** **(μS cm**^**−1**^**)**	**OC%**
NC	8.02 ^d^	145.68 ^b^	1.49 ^b^
PC	8.01 ^d^	108.82 ^a^	0.55 ^a^
TR	7.84 ^b^	110.24 ^a^	0.70 ^a^
PH	7.95 cd	105.06 ^a^	0.61 ^a^
BAC	7.92 ^bcd^	99.20 ^a^	1.15 ^ab^
AZO	7.69 ^a^	227.48 ^c^	0.49 ^a^
HFA	7.85 ^ab^	117.64 ^a^	0.56 ^a^
*Significance*	*p* < 0.001***	*p* < 0.001***	*p* = 0.039*

^1^ Treatments are as follows: *NC*, Uncontaminated soil from Velestino; *PC*, Contaminated soil from Lavrio; *TR*, Trichoderma-added Lavrio soil; *PH*, Phosbactin-added Lavrio soil; *BAC*, Bacillus-added Lavrio soil; *AZO*, Azospir-added Lavrio soil; *HFA*, Humic and fulvic acids-added Lavrio soil.

Values are the mean (*n* = 5 replicates). Different small letters within columns denote significant differences at the level of *p* < 0.005 according to Duncan’s Multiple Range Test (DMRT)

^*^Significant at the level of *p* < 0.05

^***^Significant at the level of *p* < 0.001

### Potentially toxic elements in soil

The studied soil from Lavrio, Greece (PC), was heavily contaminated with PTEs in contrast to the soil of Velestino (NC) (Table 2). The concentration of Cd in the PC treatment was 85.59 mg kg^−1^, while that of Pb was 19,232.70 mg kg^−1^, Cu = 70.84 mg kg^−1^, and Zn = 15,663.04 mg kg^−1^. As for the NC treatment, it had much lower concentrations (Cd = 2.07 mg kg^−1^, Pb = 80.47 mg kg^−1^, Cu = 42.45 mg kg^−1^; and Zn = 89.03 mg kg^−1^). The application of Phosbactin (PH) and Bacillus (BAC) resulted in a significant decrease of Cu content compared to PC (5.43 mg kg^−1^ and 57.79 mg kg^−1^, respectively), whereas the rest of the biostimulants (*Trichoderma* sp. (TR), Azospir (AZO) and Humic and Fulvic Acids (HFA)) did not influence PTE pseudo-total concentration.

**Table 2:** The effect of the studied biostimulants on the pseudo-total concentrations of Cd, Pb, Cu and Zn (mg kg^−1^) in soil

	Pseudo-total Cd (mg kg^−1^)	Pseudo-total Pb (mg kg^−1^)	Pseudo-total Cu (mg kg^−1^)	Pseudo-total Zn (mg kg^−1^)
NC	0.82 ^a^	80.47 ^a^	42.45 ^a^	89.03 ^a^
PC	85.59 ^b^	19232.70 ^b^	70.84 ^d^	15663.04 ^b^
TR	82.96 ^b^	19428.86 ^b^	65.61 ^bcd^	16607.29 ^b^
PH	82.18 ^b^	18337.33 ^b^	65.43 ^b^	15248.29 ^b^
BAC	85.08 ^b^	17238.05 ^b^	57.79 ^bc^	16741.13 ^b^
AZO	83.01^b^	17356.55 ^b^	69.47 ^cd^	16597.98 ^b^
HFA	86.32 ^b^	17986.32 ^b^	61.58 ^bcd^	15645.15 ^b^
*Significance*	*p* < 0.001***	*p* < 0.001***	*p* < 0.001***	*p* < 0.001***
BG^1^	0.41	27	38.9	70
PV (medium soil)^1^	1	70	40	150
TAV^3^	2–20	50–300	60–500	200–1500
80568/4225/91^4^	1–3	50–300	50–140	150–300

Treatments are as follows: *NC*, Uncontaminated soil from Velestino;, *PC*, Contaminated soil from Lavrio;, *TR*, *Trichoderma* sp.-added Lavrio soil;, *PH*, Phosbactin-added Lavrio soil;, *BAC*, *Bacillus* sp.-added Lavrio soil, *AZO*, Azospir-added Lavrio soil;, *HFA*, Humic and fulvic acids-added Lavrio soil. Values are the mean (*n* = 5 replicates). Different small letters within columns denote significant differences at the level of *p* < 0.05 according to Duncan’s Multiple Range test (DMRT)

***Significant at the level of p < 0.001

^1^Background level as reported by Kabata - Pendias (2011, p. 41)

^2^Precautionary value for medium-textured soil according to the German guidelines

^3^Trigger Action value as reported by Kabata - Pendias (2011, p. 24)

^4^Maximum allowable concentration of PTEs in soils where sludge application is permitted, according to the Greek Ministerial Decision 80568/4225/91

**Fig. 1:**
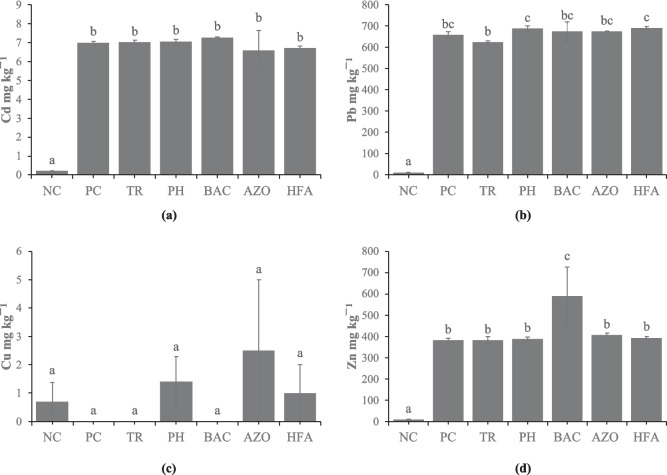
Contents of PTEs in the soil extractions with DTPA in the treatments of the five biostimulant products. (**a**) Cd, (**b**) Pb, (**c**) Cu, (**d**) Zn. Treatments are as follows: NC (Uncontaminated soil from Velestino), PC (Contaminated soil from Lavrio), TR (*Trichoderma* sp.-added Lavrio soil), PH (Phosbactin-added Lavrio soil), BAC (*Bacillus* sp.-added Lavrio soil), AZO (Azospir-added Lavrio soil), HFA (Humic and fulvic acids-added Lavrio soil). Values are the mean ± standard error (*n* = 5 replicates). Different small letters within columns denote significant differences at the level of *p* < 0.05

Soil extractions with DTPA for Cd was 0.20 mg kg^−1^ and 6.99 mg kg^−1^ for the NC and PC treatments, respectively (significantly different at *p* < 0.05). However, no significant differences were observed between the soils treated with biostimulants and the PC treatment (Fig. 1). The bioavailable fraction of Pb in soil showed that the soil of Velestino (NC) recorded significantly lower values (11.41 mg kg^−1^) compared to all the other treatments, while the TR treatment differed significantly only from PH and HFA. Moreover, the bioavailable Pb in the PC treatment was 657.76 mg kg^−1^, while none of the tested biostimulants changed the DTPA-extractable Pb compared to the PC. Additionally, the bioavailable Cu in the Velestino soil was 0.69 mg kg^−1^_,_ whereas no Cu was detected in the soil of Lavrio (PC). In contrast to that, bioavailable Cu was detected in the treatments of PH, AZO and HFA (1.41, 2.50 and 1.00 mg kg^−1^, respectively). The DTPA-extractable Zn had its highest value in the BAC treatment (589.92 mg kg^−1^), and exhibited significant variations among the rest of the biostimulants and both the controls, while the NC treatment recorded the lowest content of Zn at NC (9.06 mg kg^−1^).

### Plant growth and physiological parameters

The height of* S. oleraceus* plants was 21.63 cm in the Velestino soil (NC),significantly higher compared to the rest of the treatments (Table 3). Moreover, the application of *Bacillus* sp., Azospir and Humic and Fulvic Acids in the contaminated Lavrio soil resulted in shorter plants (11.57, 10.47, 11.75 cm, respectively) compared to the untreated contaminated soil (PC), while the heights at PH and TR were without differences from PC. The NC treatment showed the largest rosette diameter (59.69 cm), while no significant differences were observed among the rest of the treatments. Additionally, NC exhibited the highest aboveground fresh (15.50 g) and dry weight (1.01 g), as well as the highest root and leaf biomass (0.26 g and 12.49 g, respectively) and number of leaves (16). For all the growth parameters mentioned above, the added biostimulants did not show any significant differences compared to the PC, except for HFA which resulted in the lowest number of leaves (9).

**Table 3:** Plant growth parameters of *Sonchus oleraceus* plants

	Height (cm)	Rosette Diameter (cm)	Shoots FW (g)	Shoots DW (g)	Roots (g)	Number of Leaves	Weight of Leaves (g)
NC^1^	21.63 ^d^	56.69 ^b^	15.50 ^c^	1.01 ^c^	0.26 ^c^	16 ^c^	12.49 ^d^
PC	15.92 ^c^	19.86 ^a^	6.14 ^ab^	0.38 ^ab^	0.10 ^ab^	11 ^b^	4.79 ^abc^
TR	14.81 ^c^	23.76 ^a^	7.24 ^b^	0.47 ^b^	0.19 ^bc^	11 ^b^	6.31 ^c^
PH	13.69 ^abc^	21.71 ^a^	5.57 ^ab^	0.37 ^ab^	0.12 ^ab^	10 ^ab^	4.67 ^abc^
BAC	11.57 ^ab^	23.85 ^a^	6.21 ^ab^	0.41 ^ab^	0.19 ^bc^	10 ^ab^	5.49 ^bc^
AZO	10.47 ^a^	21.01 ^a^	4.64 ^a^	0.28 ^a^	0.06 ^a^	10 ^ab^	4.16 ^ab^
HFA	11.75 ^ab^	19.57 ^a^	4.05 ^a^	0.25 ^a^	0.18 ^bc^	9 ^a^	3.39 ^a^
*Significance*	***	*	***	***	***	***	***

^1^Treatments are as follows: *NC*, Uncontaminated soil from Velestino; *PC*, Contaminated soil from Lavrio; *TR*, *Trichoderma* sp.-added Lavrio soil; *PH*, Phosbactin﻿-added Lavrio soil; *BAC*, *Bacillus* sp.﻿-added Lavrio soil; *AZO*, Azospir﻿-added Lavrio soil; *HFA*, Humic and fulvic acids﻿-added Lavrio soil. Values are the mean (*n* = 15 replicates). Different small letters within columns denote significant differences at the level of *p* < 0.05

^*^Significant at the level of *p* < 0.05

^***^Significant at the level of *p* < 0.001

As shown in Table 4, no significant variations were observed in total chlorophyll content (SPAD index) in most of the treatments, with values ranging from 19.16 to 23.19 across all treatments. However, NC treatment was significantly different from PC, PH and HFA. As for the leaf area of *S. oleraceus*, it had its maximum value at NC (356.82 cm^2^), being significantly different from the rest of the treatments, while the lowest overall value was recorded in the HFA treatment (116.99 cm^2^).

**Table 4:** Plant growth parameters (leaf area, specific leaf area, SPAD index) as affected by PTEs and biostimulant application in *Sonchus oleraceus* plants

	SPAD index	Leaf Area (cm^2^)	Specific Leaf Area (m^2^ kg^−1^)
NC^1^	23.59 ^b^	356.82 ^c^	36.68 ^a^
PC	19.95 ^a^	152.09 ^ab^	50.89 ^ab^
TR	20.63 ^ab^	185.13 ^b^	41.09 ^ab^
PH	19.82 ^a^	163.25^ab^	47.75^ab^
BAC	20.32^ab^	162.05^ab^	40.26^ab^
AZO	21.48 ^ab^	144.09 ^ab^	55.57 ^b^
HFA	19.16 ^a^	116.99 ^a^	48.43 ^ab^
*Significance*	*p* = 0.097^NS^	*p* < 0.001***	*p* = 221^NS^

^1^Treatments are as follows: Uncontaminated soil from Velestino; PC, Contaminated soil from Lavrio; TR, Trichoderma sp.-added Lavrio soil; PH, Phosbactin﻿-added Lavrio soil; BAC, Bacillus sp.﻿-added Lavrio soil; AZO, Azospir﻿-added Lavrio soil; HFA, Humic and fulvic acids﻿-added Lavrio soil. Values are the mean ± standard error (n = 15 replicates). *NC*, Uncontaminated soil from Velestino; *PC*, ontaminated soil from Lavrio; *TR*, *Trichoderma* sp.-added Lavrio soil; *PH*, Phosbactin﻿-added Lavrio soil; *BAC*, *Bacillus* sp.﻿-added Lavrio soil; *AZO*, Azospir﻿-added Lavrio soil; *HFA*, Humic and fulvic acids﻿-added Lavrio soil. Values are the mean (*n* = 15 replicates). Different small letters within columns denote significant differences at the level of *p* < 0.05

^***^Significant at the level of *p* < 0.001

*NS*, non-significant

### PTE concentrations in plant tissues

**Fig. 2:**
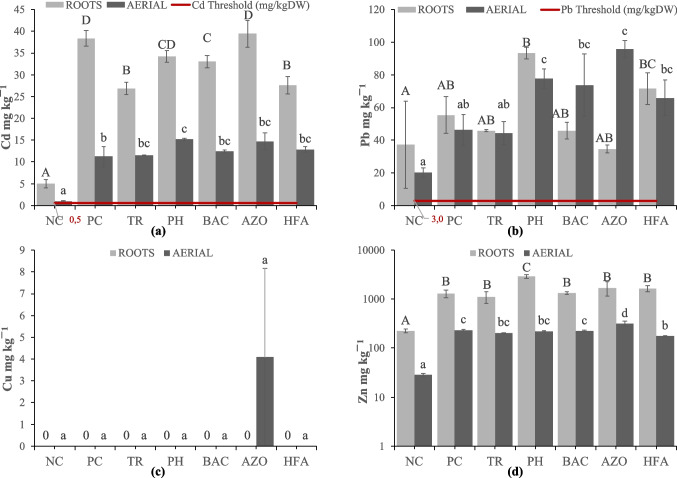
PTE concentrations of (**a**) Cd, (**b**) Pb, (**c**) Cu, and (**d**) Zn (mg kg^−1^) in plant tissues of *Sonchus oleraceus* across different treatments. Treatments are as follows: NC (uncontaminated soil from Velestino), PC (contaminated soil from Lavrio), TR (*Trichoderma* sp.﻿-added Lavrio soil), PH (Phosbactin﻿-added Lavrio soil), BAC (*Bacillus* sp.﻿-added Lavrio soil), AZO (Azospir﻿-added Lavrio soil), HFA (Humic and fulvic acids﻿-added Lavrio soil). Values are the mean ± standard error (*n* = 5 replicates). Different capital letters indicate significant differences among the treatments in roots at *p* < 0.05. Different small-case letters indicate significant differences among the treatments in the aerial parts at *p* < 0.05. “0” indicates concentration of PTEs below the detection limit (BDL). The red line represents the maximum allowable concentration for fresh vegetables, as defined by EU Regulation (EC No 1881/2006). Given an assumed dry matter content of 10%, the threshold (originally reported per fresh weight) has been adjusted to a dry weight by multiplying the value by a factor of 10

The concentration of Cd in roots of *S. oleraceus* was significantly higher at PC (38.35 mg kg^−1^) and AZO (39.42 mg kg^−1^), followed by BAC (33.05 mg kg^−1^), TR (26.88 mg kg^−1^), and HFA (27.61 mg kg^−1^), while that at NC was the lowest (5.06 mg kg^−1^). Moreover, PC showed significant difference compared to NC, TR, BAC and HFA (Fig. 2A). Lead (Pb) in roots ranged from 34.58 mg kg^−1^ (AZO) to 93.29 mg kg^−1^ (PH), with NC and PC values being 37.27 mg kg^−1^ and 55.32 mg kg^−1^, respectively (Fig. 2B). However, no significant differences were observed among any of the treatments. As for Cu concentration in roots, it was not detected in any treatment (Fig. 2C). The highest concentration of Zn in roots was observed at PH (2911.99 mg kg^−1^), being significantly different from the rest of the treatments (Fig. 2D). Zinc in roots in the non-contaminated soil was 224.76 mg kg^−1^ (the lowest overall value), while all the other treatments showed non-significant differences. Regarding the Cd content in the aerial parts of sowthistle, it was 0.99 mg kg^−1^ at NC and 11.25 mg kg^−1^ at PC, while it exhibited a significant increase at PH (15.18 mg kg^−1^). All the other treatments showed non-significant differences from PC or PH (Fig. 2A). The concentration of Pb in aerial biomass was 20.25 mg kg^−1^ at NC and 46.36 mg kg^−1^ at PC with non-significant differences between them (Fig. 2B). A significant increase was observed only at PH and AZO (77.69 mg kg^−1^ and 95.83 mg kg^−1^, respectively). Copper (Cu) was only detected at AZO (4.08 mg kg^−1^) (Fig. 2C). As for Zn, its concentration at NC was significantly lower (28.33) compared to NC (281.36) (Fig. 2D). The highest content of Zn was observed at AZO (315.87 mg kg^−1^), while the lowest was found at HFA (175.67 mg kg^−1^).

### Indices for the bioaccumulation and translocation of PTEs

Significant variations in bioaccumulation were observed for Cd, Pb and Zn (*p* < 0.001), but not for Cu (BAF = 0) (Table 5). Specifically, Cd exhibited elevated accumulation across all the treatments, with values ranging from 1.620 (PC) to 5.831 (NC). Lead BAF ranged from 0.071 (TR) to 1.760 (NC). Zinc also demonstrated significant differences in accumulation among treatments, with values ranging from 0.444 (BAC) to 3.221 (NC). The accumulation of all the studied PTEs was significantly lower in all the treatments compared to NC. When comparing the different metals in the same treatment, Cd was found to have the highest BAF value, followed by Zn and Pb, which had the lowest BAF. Regarding the translocation factor, Pb was the only metal that surpassed the critical value of TF > 1, especially in the NC, BAC and AZO treatments (1.491, 1.278, 2.817, respectively), with the latter exhibiting significant differences among all the treatments. As for Cd and Zn, they had relatively low translocation across the treatments, with TF values ranging from 0.266 (PC) to 0.471 (HFA) for Cd, and 0.076 (PH) to 0.217 (AZO) for Zn. Copper (Cu) exhibited no translocation (TF = 0). Among metals within the same treatment, TF for Cd and Zn had no significant differences except for the cases of PH and HFA. In these treatments, Cd exhibited higher TF than Zn.

**Table 5:** Bioaccumulation Factor (BAF) and Translocation Factor (TF) of the four studied PTEs (Cd, Pb, Cu, Zn) in *Sonchus oleraceus* plants

		NC^1^	PC	TR	PH	BAC	AZO	HFA	***Sig***
**BAF**	Cd	5.831 ^b**C**^	1.620 ^a**C**^	1.645 ^a**D**^	2.158 ^a**D**^	1.711 ^a**C**^	2.722 ^a**B**^	1.911 ^a**C**^	^**^
	Pb	1.760 ^b**AB**^	0.072 ^a**A**^	0.071 ^a**B**^	0.113 ^a**B**^	0.113 ^a**A**^	0.142 ^a**A**^	0.095 ^a**A**^	^*^
	Cu	0 ^**A**^	0^**A**^	0 ^A^	0 ^**A**^	0 ^**A**^	0 ^**A**^	0 ^**A**^	-
	Zn	3.221 ^b**B**^	0.610 ^a**B**^	0.541 ^a**C**^	0.569 ^a**C**^	0.444 ^a**B**^	0.789 ^a**A**^	0.447 ^a**B**^	^***^
	*Sig*	^***^	^***^	^***^	^***^	^***^	^***^	^***^	
**TF**	Cd	0.291 ^a**A**^	0.266 ^a**AB**^	0.432 ^a**B**^	0.439 ^a**B**^	0.375 ^a**B**^	0.369 ^a**A**^	0.471 ^a**B**^	^NS^
	Pb	1.491 ^a**B**^	0.842 ^a**B**^	0.849 ^a**C**^	0.923 ^a**C**^	1.278 ^a**C**^	2.817 ^b**B**^	0.776 ^a**C**^	^**^
	Cu	0 ^**A**^	0 ^**A**^	0 ^**A**^	0 ^**A**^	0 ^**A**^	0 ^**A**^	0 ^**A**^	-
	Zn	0.127 ^a**A**^	0.197 ^a**AB**^	0.275 ^a**B**^	0.076 ^a**A**^	0.168 ^a**A**^	0.217 ^a**A**^	0.109 ^a**A**^	^NS^
	*Sig*	^***^	0.065^NS^	^***^	^***^	^***^	^***^	^***^	

^1^Treatments are as follows: *NC*, Uncontaminated soil from Velestino; *PC*, contaminated soil from Lavrio; *TR*, *Trichoderma* sp.﻿-added Lavrio soil; *PH*, Phosbactin﻿-added Lavrio soil; *BAC*, *Bacillus* sp.﻿-added Lavrio soil; *AZO*, Azospir﻿-added Lavrio soil; *HFA*, Humic and fulvic acids﻿-added Lavrio soil. Values are the mean (*n* = 15 replicates). Different small-case letters denote significant differences among treatments at *p* < 0.05 within the same line. Different capital letters indicate significant differences among treatments at *p* < 0.05 within the same column

^***^Significant at the level of *p* < 0.001

^**^Significant at the level of *p* < 0.01

*NS*, Non-significant

## Discussion

### Effects of biostimulants on soil properties

Regarding the pH values of the two control treatments (NC and PC), they were relatively high, indicating the alkaline nature of the tested soils. The decline of pH in soils treated with TR, AZO and HFA demonstrate that these amendments can effectively reduce soil alkalinity. Specifically, the AZO treatment led to the lowest pH value (7.69), which may be attributed to the capacity of *Azotobacter* spp. to produce organic acids such as lactic acid, formic acid and acetic acid that consequently lead to pH reduction. These organic acids can lead to CaCO_3_ solubilization of the two calcareous soils, thereby resulting in the lowering of the alkalinity of the soil environment (Rashad et al. 2023). The slight decrease in pH in soils treated with TR and HFA also aligns with previous studies indicating that these amendments may improve soil health by modulating pH values and elevated micronutrient availability (Conte et al. 2022; Liu et al. 2024). In terms of EC, none of the soil amendments showed a significant variation from PC except for the AZO treatment, which showed a significant increase in EC (227.48 μS cm^−1^). This value does not constitute in any way a concern for plant growth since it is rather low. The stable organic carbon content in all the treatments, except for the higher initial value at NC, indicates that amendments did not lead to significant changes in OC levels within the short period that our experiment was carried out.

### Role of biostimulants in modulating soil PTE availability

This study also highlights the significant difference among biostimulants in response to various PTE levels in soil. As expected, the studied soil underscores the severe contamination in Lavrio (used as positive control) and the relatively uncontaminated nature of soil in Velestino (used as negative control). The elevated concentrations of pseudo-total Pb and Zn in particular are indicative of anthropogenic impacts of the smelting and mining activities performed in the Lavrio region in the past (although they now are discontinued) (Apostolopoulos 2014). The pseudo-total concentration of Cd was 85.59 mg kg^−1^, significantly higher than the corresponding background level of 0.41 mg kg^−1^ (Kabata-Pendias 2011, p. 41) and far exceeded the regulatory threshold of 1–3 mg kg^−1^ set for soils where sludge application is to be applied (Greek Ministerial Decision 1991). Similar findings were reported in a previous study by Thalassinos et al. (2023a), which recorded a concentration of 101.87 mg kg^−1^, confirming the severity of contamination in the area. Our findings also show that biostimulants have different effects on the bioavailability of PTEs in the tested contaminated soil. All studied DTPA-extractable PTEs were significantly higher in the contaminated soil compared to Velestino (NC), due to the differences in the nature of soils. Interestingly, none of the biostimulants had any significant effect on Cd and Pb bioavailabity. Contrary to our findings, previous studies have suggested that specific biostimulants can affect the bioavailability of PTEs. For instance, humic substances may have complex interactions with PTEs like cationic metals, as organic acids can dissolve metals and elevate their availability in soil (Evangelou et al. 2004). A study by Grammenou et al. (2024) showed that the application of HFA in sowthistle plants increased the Cd-DTPA levels. On the other hand, bioavailable Zn showed great variations across the treatments, with the highest concentration being recorded in the BAC treatment (589.92 mg kg^−1^). Our results are in agreement with several other studies that have shown that *Bacillus* species can influence Zn availability (Masood et al. 2022). It is likely that bacterial strains such as *Bacillus aryabhattai* and *B. subtilis* may have dissolved previously insoluble Zn into soluble forms, enhancing its bioavailability in soil (Mumtaz et al. 2017). Bacteria are known to enhance Zn solubility through various mechanisms. These include the chelation of Zn and Fe from siderophores produced by these bacteria, changes in soil pH, the biosynthesis of phytohormones such as auxin and ethylene, and the production of organic acids (e.g., lactic acid, butyric acid, and oxalic acid) (Fasim et al. 2002). These organic acids bind cations and improve their availability by lowering pH (Masood et al. 2022). Regarding the DTPA-extractable Cu, it was not detected in the soil of Lavrio, and this absence may indicate a disparity in contamination profile or in the detection limits of the analytical methods employed. However, biostimulants derived from microorganisms like *Azotobacter* sp., *Azospirillum* sp. and *Bacillus* sp. were found to have an impact on the detection of Cu. This phenomenon may be attributed to the production of siderophores (Fe-complexing molecules) by microorganisms in the soil, which promote the production of soluble complexes and the binding of copper ions, thus enhancing their availability and detectability in the soil (Chibuike and Obiora 2014). On the other hand, in the absence of biostimulants, the natural availability of Cu may be limited due to the inadequate siderophores required for Cu fixation in soil. The efficacy of biostimulants may be contingent upon the soil properties and the biostimulants used. Consequently, in heavily contaminated soils a more targeted approach may be necessary with several remediative tools being implemented.

### Influence of treatments on PTE uptake by *Sonchus oleraceus*

Previous studies have demonstrated the capability of sowthistle to accumulate various PTEs (Xiong 1997; Samadi et al. 2019; Grammenou et al. 2024). Based on this criterion, species was selected as a test plant for phytoremediation purposes in our study. The results demonstrate that *S. oleraceus* can accumulate elevated amounts of PTEs within its shoots and roots. The presence of Cd in plant roots were significantly higher in plants grown in the PC treatment (38.35 mg kg^−1^), reflecting the increased level of DTPA-extractable Cd in soil compared to NC. However, the application of TR, BAC and HFA effectively reduced the Cd concentration in roots, despite the high bioavailability of Cd in soil. *Trichoderma harzianum* did not affect the bioavailability of PTEs in the soil, but was associated with a reduction in root Cd accumulation. This finding contradicts previous studies, according to which *T*. *harzianum* promoted the translocation and accumulation of both Cd and As in *Brassica juncea* plant tissues (Yao et al. 2023). Furthermore, *T. harzianum* TS143 appeared to mainly enhance Cd accumulation in roots and shoots of maize, with a greater increase in shoots (Pehlivan et al. 2021). Like other fungal species, *Trichoderma* genus can adsorb, transform, and biomineralize PTEs in the environment. Both live and dead fungal mycelia interact with PTEs, influencing their mobility and bioavailability (Luo et al. 2022). Fungal mycelia can bind PTEs through the functional groups found in their cell walls, which consist of polysaccharides, proteins and lipids. However, under specific circumstances (e.g., pH variations, presence of certain chemical compounds, decomposition of fungal biomass), these PTEs may be reintroduced into the environment. Therefore, fungi can temporarily store PTEs, reducing their bioavailability, but then release them, making them available again (Kurek and Majewska 2004). The other biostimulants (BAC, HFA) may induce plant detoxification mechanisms, including the increased production of metallothioneins (MTs) glutathione (GSH), phytochelatins (PCs) or other chelating compounds that bind Cd in plant tissues, thus reducing its bioaccumulation (Bartucca et al. 2022). Cadmium accumulation was also high in the aerial biomass in the PC (11.25 mg kg^−1^) compared to NC (0.99 mg kg^−1^). Cd uptake exceeded the maximum allowable concentration for fresh vegetables, as defined by EU Regulation (EC No 1881/2006) established by the Commission of the European Communities (CEC 2006), which sets a limit of 0.05 mg kg^−1^ fresh weight. Given that the moisture was assumed to be 90%, the threshold was adjusted to a dry weight by multiplying the value by 10. Similar limits have been set by China through National Food Safety Standard of Maximum Levels of Contaminants in Foods (GB 2762–2012), which regulates the maximum levels of contaminants in food, including Cd. It is noteworthy that the expected levels of Cd in plants cultivated in pristine soils are nearly zero (Han et al. 2023). Among the different biostimulants used, PH resulted in a notable increase in Cd contents within the aerial parts. Phosbactin contains various strains of *B. megaterium*, thus our findings align with the results of Li et al. (2021), who reported that *B. subtilis* inoculation induced a significant elevation of Cd in the aerial biomass of *Medicago sativa* L. Similar findings were reported by Esringü et al. (2014), whose results also concur with our findings: They observed a significant increase in Cd content in *Brassica napus* treated with *B. megaterium.* As for Pb, despite the high concentrations in the roots, no significant changes were observed among the biostimulant applications. Comparable results to Cd were observed for Pb accumulation in the aboveground plant tissues, whose levels surpassed the permissible limits for fresh vegetables set by the European Union (0.3 mg kg^−1^ FW, EU Regulation (EC No 1881/2006; CEC 2006), China (0.1 mg kg^−1^ FW National Food Safety Standard (GB 2762–2012)), and Australia (0.1 mg kg^−1^ FW Standard 1.4.1 – Contaminants and Natural Toxicants). In the aerial biomass, significant increases were detected at PH (77.69 mg kg^−1^) and AZO (95.83 mg kg^−1^) compared to PC. Biostimulants derived from plant growth promoting rhizobacteria (PGPR) and free-living soil bacteria, such as *Azotobacter* sp., *Bacillus* sp. or *Rhizobium* sp., may promote and improve plant vigor in contaminated soils by reducing harmful impacts of PTEs on plants (Tiwari et al. 2013). In the same line, He et al. (2020) reported that PGPR inoculation increased Cd and Pb content in shoots and roots of *Solanum nigrum.* Regarding Cu levels in plant tissues, they were not detected in either roots or shoots, except for a small amount in the AZO treatment (4.08 mg kg^−1^), possibly due to its lower availability. The highest concentration of Zn in roots was found at PH (2911.99 mg kg^−1^), significantly different from the PC treatment (1303.93 mg kg^−1^). The lower levels of Zn in the uncontaminated soil (224.76 mg kg^−1^) exhibited the capacity of *S. oleraceus* to accumulate Zn in contaminated environments. Zinc concentration was higher in the aerial biomass at AZO (315.87 mg kg^−1^) and the lowest level was recorded at HFA (175.67 mg kg^−1^). Similar results were reported in the research of Jha et al. (2017), who recorded the accumulation of significant amounts of Zn and Pb in the leaves of maize inoculated with PGPR isolates. At the same time PGPR provide additional support to plant survival when exposed to PTEs. They also reported that the reduction in PTE concentration was directly related to the increase in the dosage of humic substances. Overall, humic acids are high molecular weight organic acids (HMWOAs) that can influence the mobility of PTEs at the soil–plant interface. These organic components can mitigate the mobility of PTEs by forming chelating bonds with metal ions, thus mitigating their accumulation by plants and their transport through the food chain (Grammenou et al. 2024). However, for a plant to qualify as a hyperaccumulator, its shoots must contain > 100 mg Cd kg^−1^, > 1000 mg Pb and Cu kg^−1^, or > 10,000 mg Zn kg^−1^ (dry weight), when grown in soils enriched with toxic elements (Del Río-Celestino et al. 2006).

### Plant growth responses to biostimulants under elevated PTE concentrations

Despite the significant increase of PTE levels in *S. oleraceus* plants after the addition of the studied biostimulants, plant growth parameters, e.g., number of leaves, and roots and shoots biomass, appeared to remain unchanged. This indicates the development of defense mechanisms by *S. oleraceus* plants after exposure to high concentrations of toxic components (Narayanan and Ma 2023). Alternatively, the potential negative effects of elevated PTEs were offset by the beneficial effects of biostimulants. The exception for this is plant height, which appears to have been significantly reduced in all biostimulant treatments. This could be attributed to changes in hormonal balance or to the fact that Lavrio soil was not appropriate for *S. oleraceus* cultivation since the plants grown in PC also recorded reduced plant height compared to NC.

### Assessment of PTE mobility in plants through the BAF and TF indices

The potential of a plant for phytoremediation is typically assessed based on key indices, e.g., the bioaccumulation factor (BAF) and the translocation factor (TF). Plants having these two indices with values higher than 1 are considered suitable candidates for phytoextraction (Sharma et al. 2023). Cadmium showed the highest bioaccumulation, with BAF values ranging from 1.620 (PC) to 5.831 (NC). This suggests that Cd is accumulated to a significant extent in plant tissues regardless of treatment conditions; this may be due to its high availability and strong absorption capacity. Despite the significant bioaccumulation, the translocation factor (TF) values for Cd remained below 1 in all treatments, ranging from 0.266 (PC) to 0.471 (HFA). This means that Cd was mainly confined to the roots and was not efficiently transported to the upper parts of the plant, limiting the possibility of toxicity to aboveground tissues. This contradicts the findings reported by Grammenou et al. (2024), who identified sowthistle as a potential Cd hyperaccumulator and reported TF values much higher than 1 in a spiked soil, where bioavailable Cd concentrations were 45 mg kg^−1^. This difference could be due to differences in the physicochemical properties of soils tested. Lead showed variation in BAF values, ranging from 0.071 (TR) to 1.760 (NC). The higher Pb accumulation in NC may be related to conditions that increase the availability of Pb in the soil, despite the initial low availability of the element. However, Pb stood out with high TF values under specific treatments (NC, BAC, AZO), ranging from 1.491 to 2.817, exceeding thus the critical threshold of 1. This ability of Pb to be transported from roots to leaves indicates that Pb has significant mobility within the tissues of our test plant, which may affect the quality of upper plant parts, with remarkable consequences concerning the food chain. Similar results were observed in a study conducted in a soil from a polymetallic mining site. In particular, Bech et al. (2012) found that *S. oleraceus* was effective in Pb uptake and transport, with TF = 1.8 (the soil contained 4205 mg Pb kg^−1^). Regarding Cu, it showed no bioaccumulation (BAF = 0) or transport (TF = 0). As for Zn, it showed moderate BAF values, ranging from 0.444 (BAC) to 3.221 (NC), indicating that Zn accumulation also depends on the bioavailable concentration in soil, since no treatment showed any significant difference. The moderate bioaccumulation of Zn, coupled with TF values ranging from 0.076 (PH) to 0.217 (AZO), suggests that although Zn is efficiently absorbed by roots, its translocation to the upper parts of the plant is limited. This suggests that Zn had limited mobility in our test plant, despite the fact that it is an essential micronutrient. Similarly, according to Abdelgawad et al. (2023), *S. oleraceus* was reported to have accumulated Zn and Cu in their roots, while the value of translocation factor was considered crucial as it was close to unity.

## Conclusions

Our results exhibited the complex interaction between biostimulants, the availability of PTEs in soil, and their concentration in plant tissues. The application of *Bacillus spp.* (BAC) increased Zn availability in soil without a corresponding increase in its translocation to plant tissues. In the root system, the treatment with Phosbactin (PH) led to increased Zn concentration, while in the above-ground plant tissues, *Azospirillum* (AZO) enhanced Zn concentration, in contrast to the treatment with the addition of humic and fulvic acids (HFA), which reduced it. Additionally, PH increased the concentration of Cd and Pb in aerial parts, indicating a targeted enhancement of the transport of specific elements. Bioaccumulation factor (BAF) indicated increased uptake of Cd and Zn by *S. oleraceus*, whereas Pb showed the highest mobility toward aboveground plant compartments, especially at AZO. In contrast, Cd and Zn showed limited mobility, with concentration mainly in the roots. Overall, the treatments did not have uniform efficacy; however, specific combinations, such as PH and AZO in synergy with *S. oleraceus*, demonstrated a potential for enhancing plant uptake and transport of selected PTEs. We conclude that the application of biostimulants offers significant benefits in modifying the availability and concentration of PTEs in both soil and plant tissues, indicating a promising approach for improving phytoremediation strategies in contaminated soils and increasing soil health and ecosystem services. Nevertheless, we acknowledge the need for further research under long-term and more realistic conditions.

## Supplementary information

Below is the link to the electronic supplementary material.

ESM 1 (DOCX 10.1 MB)

## Data Availability

Data will become available on reasonable request.
