# Supplementary material for: Biostimulants efficacy in growing Sonchus oleraceus plants in contaminated mining soil with potentially toxic elements (PTEs)
Source: Environ Sci Pollut Res Int. 2025 Jun 9;32(25):15374–87. doi: 10.1007/s11356-025-36577-z (PMC12202616; doi:10.1007/s11356-025-36577-z)
Supplement: Supplementary file 1 — (DOCX 10.1 MB) [file 11356_2025_36577_MOESM1_ESM.docx]

**Supplementary material of the article titled**

**Biostimulants Efficacy in Growing *Sonchus oleraceus* Plants in Contaminated Mining Soil with Potentially Toxic Elements (PTEs)**

**Aspasia Grammenou, Spyridon A. Petropoulos, Georgios Thalassinos and Vasileios Antoniadis***

University of Thessaly, Fytokou Street, 38446 Volos, Greece.

*Corresponding author. E-mail: [antoniadis@uth.gr](mailto:antoniadis@uth.gr)

Contributing authors: [fangio57gr@gmail.com](mailto:fangio57gr@gmail.com); [asgrammenou@uth.gr](mailto:asgrammenou@uth.gr); [thalassinosgeorgios@hotmail.gr](mailto:thalassinosgeorgios@hotmail.gr); antoniadis@uth.gr

**
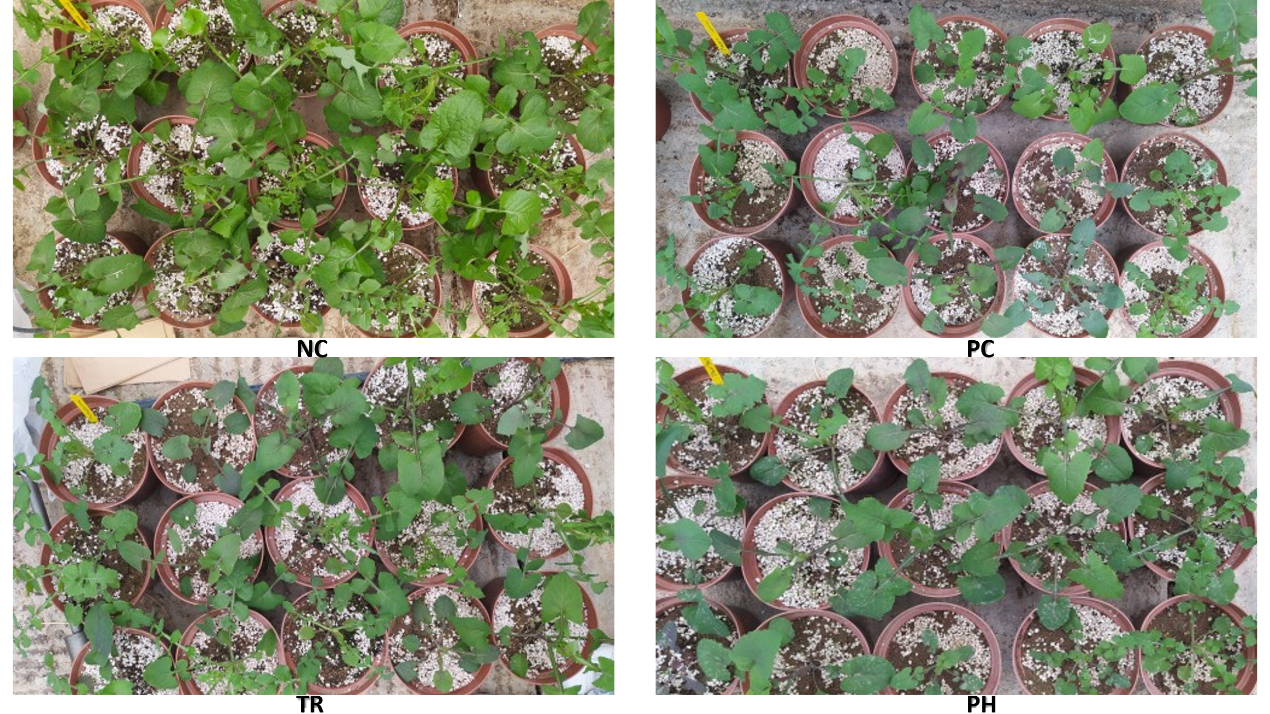
**


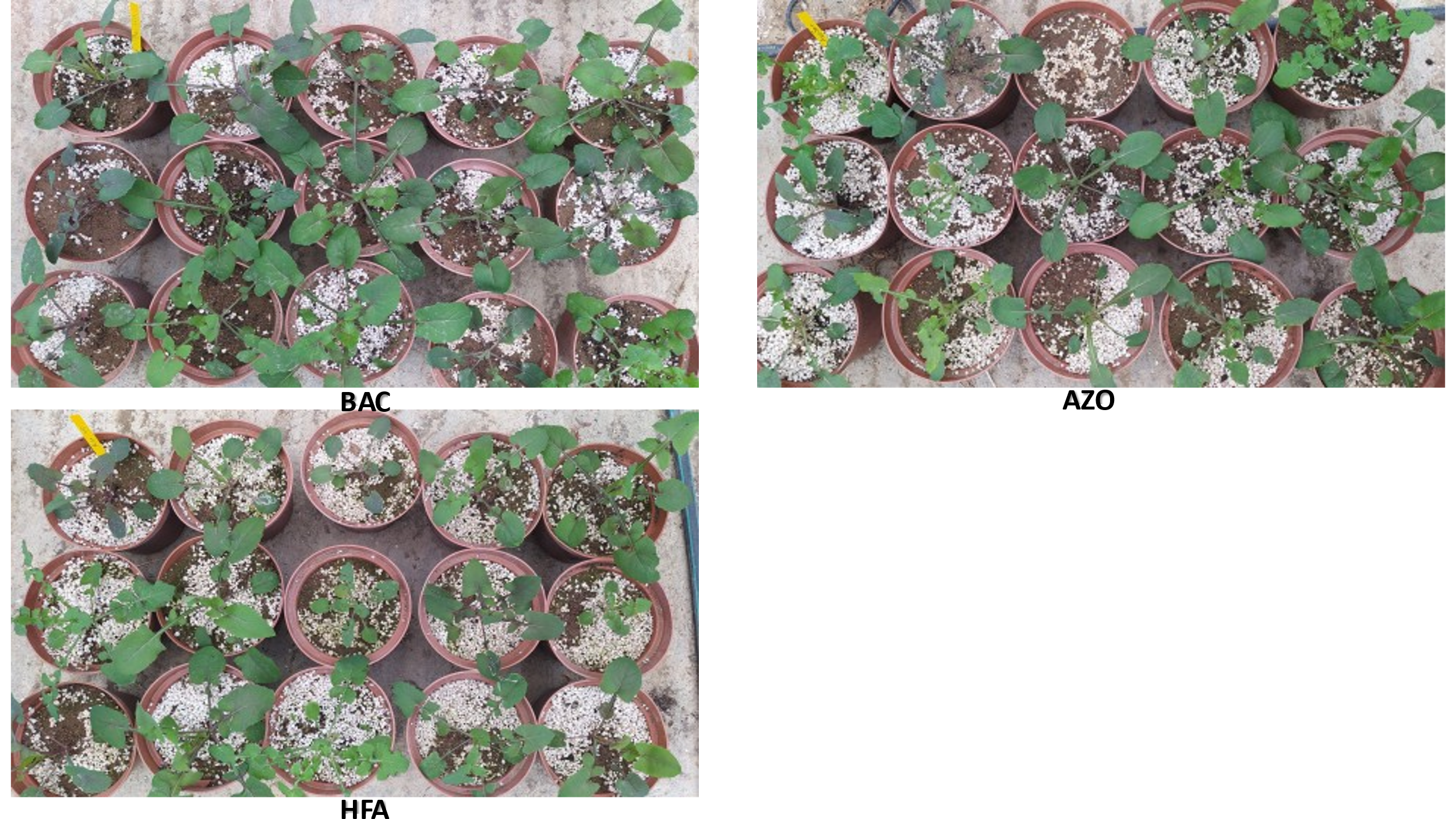


**Figure 1S.** Overview of all treatments during the experiment. Labels indicate the different treatment group; Treatments are as follows: NC (Soil from Velestino), PC (Soil from Lavrio), TR (Trichoderma sp.), PH (Phosbactin), BAC (Bacillus sp.), AZO (Azospir), HFA (Humic and Fulvic Acids).

**Table S1.** Overview of experimental treatments and application protocol.

| **Soil Type** | **Treatment Code** | **Biostimulant (if applicable)** | **Biostimulant Concentration** | **Solution preparation** | **Dosage** | **Application Method** | **Application Day(s)** | **Replicates** |
| --- | --- | --- | --- | --- | --- | --- | --- | --- |
| Non-contaminated soil (Velestino) | NC | Negative Control (no biostimulant) | - | - | - | - | - | 15 |
| Contaminated soil (Lavrio) | PC | Positive Control (no biostimulant) | - | - | - | - | - | 15 |
| Contaminated soil (Lavrio) | BAC | *Bacillus* consortium  (*B. megaerium*, *B. altitudiris*, *B. subtilis*, *B. licheniformis,* *B. methylotrophicus)* | 10^9^ cfu g^-1^ of each species. | 0.6 g in 900 mL distilled water | 3 mL/pot | Soil Drench | Transplantation. Day 15, Day 30 | 15 |
| Contaminated soil (Lavrio) | TR | Liquid solution *Trichoderma harzianum* T78 |  |  | 3 mL/Pot | Soil Drench | Transplantation. Day 15, Day 30 | 15 |
| Contaminated soil (Lavrio) | PH | *Bacillus megaterium* | 1 × 10^12^ cfu L^-^ | 0.192 mL in 1.5 L distilled water | 100 mL/Pot) | Soil Drench | Transplantation | 15 |
| Contaminated soil (Lavrio) | AZO | *Azospirillum* sp.  *Azotobacter* sp. | 1 × 10^12^ cfu L^-1^ of each species | 0.384 mL in 288 mL distilled water | 100 mL/Pot) | Soil Drench | Transplantation | 15 |
| Contaminated soil (Lavrio) | HFA | Humic and fulvic acids | 70:30 ratio derived from refined leonardite extract  OC = 4.83% | 1.1 mL in 373.9 mL distilled water | 50 mL/Pot | Root dipping + Soil drench | Transplantation, Day 5, Day 15, Day 25 | 15 |
